# Direct entry of cell-penetrating peptide can be controlled by maneuvering the membrane curvature

**DOI:** 10.1038/s41598-020-79518-1

**Published:** 2021-01-08

**Authors:** Kazutami Sakamoto, Taku Morishita, Kenichi Aburai, Daisuke Ito, Tomohiro Imura, Kenichi Sakai, Masahiko Abe, Ikuhiko Nakase, Shiroh Futaki, Hideki Sakai

**Affiliations:** 1 Tokyo University of Science, 2641 Yamazaki, Noda, Chiba 278-8510 Japan; 2 The National Institute of Advanced Industrial Science and Technology, Tsukuba, Ibaraki 305-8565 Japan; 3 Present Address: Osaka Prefecture University, Sakai, Osaka, 599-8570 Japan; 4 Kyoto University, Uji, Kyoto 611-0011 Japan

**Keywords:** Biochemistry, Biophysics, Chemical biology, Chemistry, Nanoscience and technology

## Abstract

A biomembrane's role is to be a barrier for interior cytosol from an exterior environment to execute the cell's normal biological functions. However, a water-soluble peptide called cell-penetrating peptide (CPP) has been known for its ability to directly penetrate through the biomembranes into cells (cytolysis) without perturbating cell viability and expected to be a promising drug delivery vector. Examples of CPP include peptides with multiple arginine units with strong cationic properties, which is the key to cytolysis. Here we show the conclusive evidence to support the mechanism of CPP’s cytolysis and way to control it. The mechanism we proposed is attributed to biomembrane’s physicochemical nature as lamellar liquid crystal (Lα). Cytolysis occurs as the temporal and local dynamic phase transitions from Lα to an undulated lamellar with pores called Mesh_1_. We have shown this phase transfer of Lα composed of dioleoyl-phosphatidylcholine (DOPC) with water by adding oligo-arginine (Rx) as CPP at the equilibrium. Using giant unilamellar vesicle composed of DOPC as a single cell model, we could control the level of cytolysis of CPP (FITC-R8) by changing the curvature of the membrane through osmotic pressure modulation. The cytolysis of CPP utilizes biomembrane's inherent topological and functional flexibility corresponding to the stimuli.

## Introduction

The discreetness of cells is the key to life, with bio-membrane separating interior cytosol from the environment. In this regard, cytosol is never connected to outside media, even during cell fission or fusion. However, a water-soluble peptide called cell-penetrating peptide (CPP) has been known for its penetration ability through bio-membranes without incorporating a particular transporter or receptor. Moreover, it is expected to be a potent carrier for drug delivery systems (DDS)^1–5^. There are two pathways for CPP’s internalization: direct membrane permeation to the cytosol (cytolysis) and endosome mediated uptake (endocytosis)^1^. Cytolysis is a preferable way of CPP for DDS as it can deliver drugs directly into the cytosol. On the other hand, CPP encapsulated in the endosome must cross the endosome membrane to reach the cytosol target.

Although there are many investigations reported for CPP internalization, no conclusive cytolysis mechanism is known^1–5^. Our objective here is to find a way to control cytolysis of CPP by exploring its mechanism. Our approach is to take CPP’s transmembrane penetration as a physicochemical phenomenon of bio-membrane structure and function. Bio-membrane has a lamellar bilayer structure (Lα) under the state of lyotropic liquid crystal (LC). The Lα state makes cell membranes flexible enough to accommodate cells to change shapes while keeping ordered lipid orientations as a barrier. It is generally known that LC, as a highly ordered self-organization of amphiphiles, has a series of structures such as lamellar (Lα), cubic (V_1_), and hexagonal (H_1_). The molecular geometry parameter of amphiphile (Surfactant Parameter: ***SP***, defined by Israelachvili et al.) determines each LC's topology^6–8^. The ***SP*** value corresponds to the proportion of the hydrophilic part and the hydrophobic part in the amphiphile and correlates to the surface curvature of molecular assembly as LC. The ***SP*** value is determined by the structure of amphiphile and expressed as ***SP*** = ***v***/(***a*** × ***l***) (Eq. 1), where ***a*** is the cross-sectional area per molecule at the hydrophilic-hydrophobic interface, ***l ***is the length of hydrophobic parts and ***v*** is the volume of hydrophobic parts shown as Supplementary Fig. S1.

***SP*** can be changed by ***a****, ****l,*** or ***v*** by modulating the level of ionization, hydration, solute, temperature, and many other physical (c.a. osmotic pressure) or chemical (c.a. CPP) stimulations^9^. Lα as planar lamellar layers has zero mean curvature and slight curvature modification toward positive leads to bi-continuous cubic phase (V_1_) where water and lipid are independently interconnected. This phase transition sometimes occurs through a meta-stable mesh phase (Mesh_1_), which consists of undulated bilayers with an array of pores of catenoid structure with saddle-splay negative Gaussian curvature (Fig. S1c)^8^. The phase transition from Lα to V_1_ occurs with a slight curvature change, and the energy required for this phase transition is significantly small^6–8^.

**Figure 1:**
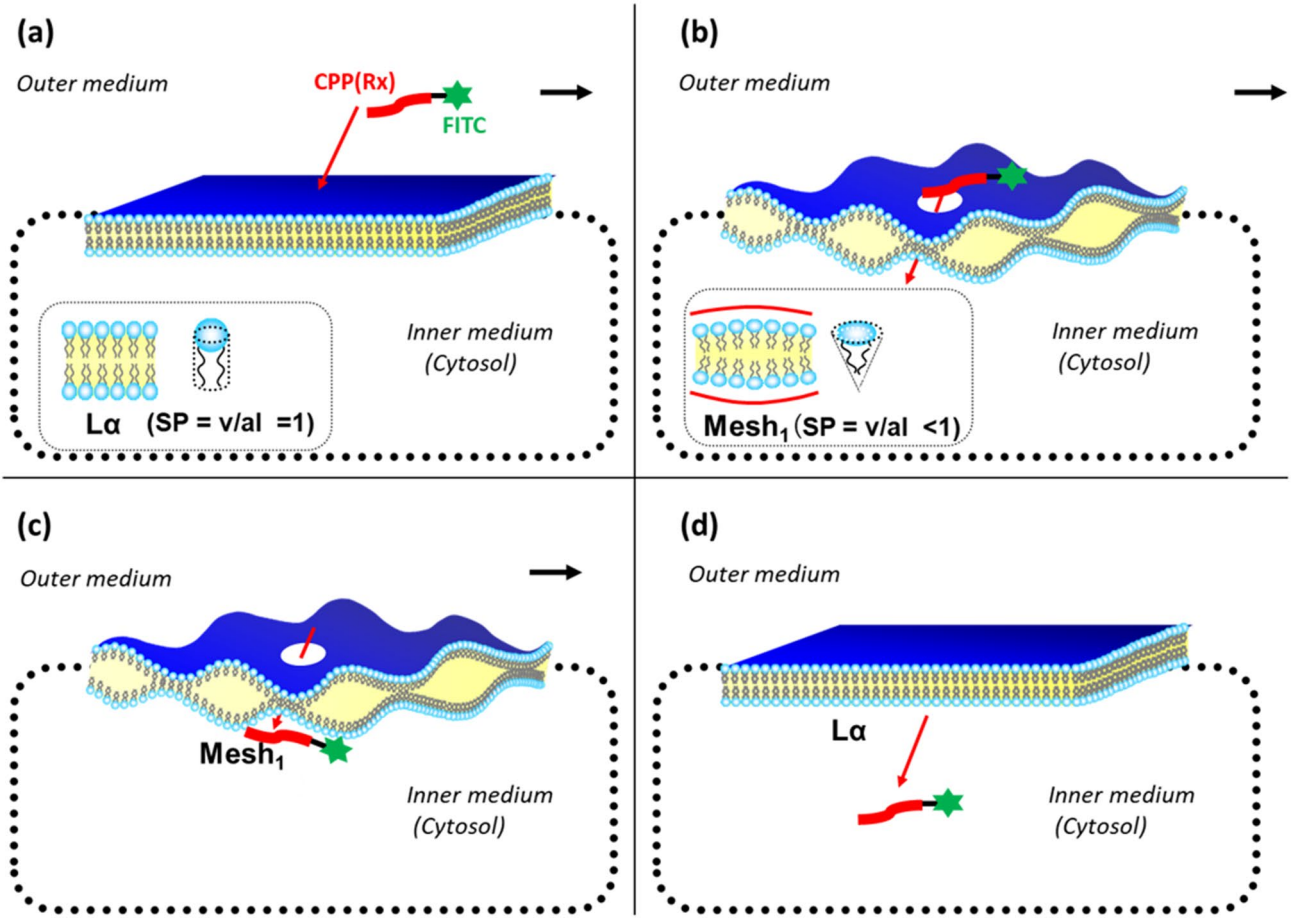
Mechanism of Cytolytic direct internalization (Cytolysis) of Origo-arginine (Rx) as CPP by Temporal and Dynamic phase transition from Lα to Mesh_1_. (**a**) Electrostatic adsorption of CPP molecule, (**b**) generation of positive curvature to induce phase transition to Mesh_1_, (**c**) translocation to the inner side of membrane under Mesh_1_, (**d**) desorption into cytosol.

Taking this into account, we have investigated the mechanism of CPP cytolysis. Our hypothesis is temporal and local dynamic phase transition from Lα to Mesh_1_ caused by electrostatic adsorption of CPP molecules generating positive curvature^10,11^ (decreasing ***SP*** by increasing ***a*** by Eq. (1)). Figure 1 shows the mechanism. CPP with condensed cationic charge come to interact with the phosphate anion of lipid membrane (Fig. 1a), then changes the membrane curvature of the contact point to more positive, which corresponds to the local and temporal topological deformation of the membrane to Mesh_1_ as a dynamic fluctuation at the contact point (Fig. 1b). Through the local pore structure of Mesh_1_, a strongly cationic CPP interacting with phosphate anion can move into the interior cytosol (Fig. 1c), then released into the cytosol, and the membrane returns to Lα (Fig. 1d). Because this phase change is local and temporal within the fluctuation of lipid molecules as LC, there would be no pores remaining afterward. This hypothesis can be illustrated by the analogy of “The Man (Mr.D) who could walk through walls” explained in Supplementary Fig. S2^12^. Namely, When Mr. D approaches the wall (CPP adsorbs to the Lα membrane), the wall surrounding him melts into a fluid (Lα turns to Mesh1), and Mr. D found himself at the other side of the wall (CPP can move into the cytosol through the temporal Mesh_1_ pore: Fig. S2); there is a hard wall behind Mr. D (the membrane then returns to Lα). If the wall (membrane) is resistant to changing its curvature, Mr. D (the CPP) will have difficulty walking through the wall (penetrating the membrane).

## Results and discussion

### Effect of oligo-arginine to induce positive curvature for membrane lipid under equilibrium condition

**Figure 2:**
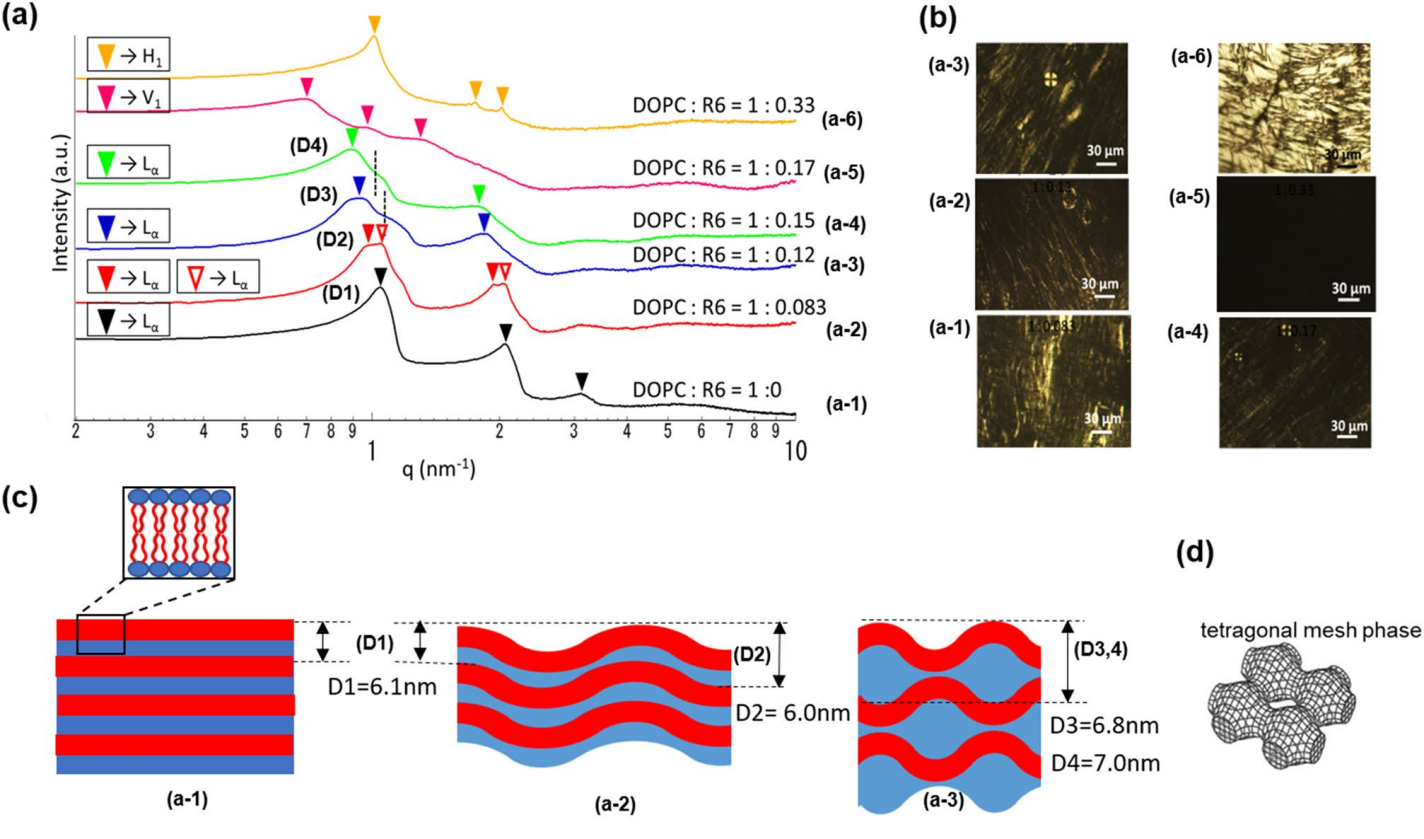
Effect of R6 to the structure of lyotropic liquid crystal composed of DOPC/Water (60/40 wt %) system at 25 °C. (**a**) Small angle Xray scattering (SAXS) spectra for DOPC/Water (60/40 wt %) system with addition of R6; (**a-1**)  , (**a-2**)  , (**a-3**)  , (**a-4**)  are Lα with q value peak ratio 1:2:3, Coexistence of V_1_ phase with the shoulder peaks were indicated by dotted vertical line for (**a-3**) and (**a-4**), (**a-5**)  is V_1_ (1:√6:√8 ), and (**a-6**)  is H_1_ (1:√3:2); (**b**) Polarized microscope image for each composition, (**c**) Schematic diagram of DOPC/Water Lα based on the results shown in (**a**,**b**). Expansion of inter layer thickness (D1) to (D2,3,4) indicates curved deformation of planar Lα structure to undulated lamellar as depicted, and D3 and D4 reasonably fit to the Mesh_1_ structure as tetragonal mesh_1_ shown in (**d**).

In order to clarify whether CPP could make membrane curvature positive by reducing SP value, we have investigated the effect of oligo-arginine (Rx) on the phase structure of LC composed of dioleoyl-phosphatidylcholine (DOPC) with water under the equilibrium condition by small-angle Xray scattering (SAXS) measurement as shown in Fig. 2. We can determine the LC structure from the SAXS peaks through the crystallographic analysis of peak ratios expressed as q values.

Theoretically, LC structure would change from lamellar (Lα) to bicontinuous cubic (V_1_) then hexagonal (H_1_) by reducing ***SP*** value or increasing curvature toward the water, and Mesh_1_ would exist in between Lα and V_1_ (Fig. S1), and along with this phase transitions, peak ratio changes from 1:2:3 for Lα and Mesh_1,_ then 1:√6:√8 for V_1_, and 1:√3:2 for H_1_ theoretically. As shown in Fig. 2a, q values of SXAS peaks for hexa-arginine (R6) added to the DOPC/Water (60/40 wt%) system followed 1:2:3 q-peak ratio as Lα within the range of DOPC: R6 molar ratio from 1:0.083 (a-2) to 1:0.15 (a-4) (Fig. 2). The V_1_ phase's coexistence in Lα was indicated for (a-3) and (a-4) by the shoulder peaks shown by vertical dotted lines. Further incremental addition of R6 changed the structure to V_1_ (1:√6:√8 ) (a-5) then to H_1_ (1:√3:2) (a-6). We also made polarized microscope observations for each sample, as shown in Fig. 2b which agree with the SAXS peak analysis. Namely, oil-streak texture for the Lα phase (a-1 to a-4), no birefringence as a cubic phase for the V_1_ (a-5), and fan-like textures for the H_1_ (a-6). These consecutive phase changes by the addition of R6 are the proof of substantial cationic property of guanidium moiety of arginine in R6 to interact with phosphate anion of DOPC to make the hydrophilic part more considerable corresponding to the larger ***a*** value (decreasing ***SP*** in Eq. (1) and increasing membrane curvature. Fig. S1).

It is noteworthy that interlayer spacing D calculated by the first peak q value increased consecutively within the Lα state (DOPC: R6 up to 1:0.15), as shown in Fig. 2a and c. Usually, such an increasing D value is observed by increasing the water ratio resulting in the interlayer water thickness larger, as shown in Supplementary Fig. S3 for a simple DOPC/Water system without Rx. Namely, interlayer spacing D increased consecutively by decreasing DOPC concentration and reached a constant level of 6.4 nm (Fig. S3b,d) at DOPC < 50 wt% with the formation of vesicles confirmed by maltase cross texture as shown in (Fig. S3c). On the other hand, R6 has added to the DOPC/Water (60/40) system in the current system (Fig. 2). Namely, the current system's water amount is fixed so that water layer expansion in the planar lamellar layers would not be possible (Fig. 2c).

We observed similar D layer expansion in Lα when R4 or R8 was added to the DOPC/Water (60/40) system. As a strong cation, Rx induces positive curvature by increasing ***a*** value in Eq. (1) under equilibrium condition. Here we assume this D value increase is attributed to the curved deformation of planar Lα structure to undulated lamellar as shown in Fig. 2c, which reasonably fits the Mesh_1_ structure as a metastable phase in between Lα and V_1_. Hyde et al. reported the Mesh_1_ phase as a punctured bilayer to accommodate increased vending energy by the slight change of ***S*** to give periodical curvatures to the planar lamellar layers. They emphasized the physiological relevance as intermediate membrane topologies^7,8^. This assumption supports our hypothesis of temporal and local dynamic phase transition from Lα to Mesh_1_, as shown in Fig. 1.

### Effect of osmotic pressure to control cytolysis

In order to establish a way to control CPP Cytolysis, we made the second hypothesis that CPP’s translocation could be controlled if the positive curvature change is stimulated or suppressed utilizing physical or chemical modulation^9^. To prove this, we first used erythrocyte (red blood cell), which has a slightly negative mean membrane curvature and an ability to change its shape by osmotic pressure corresponding to the curvature modulation. We have applied osmotic pressure within the range of no hemolysis to keep the barrier function intact. We found that erythrocyte is resistant to CPP penetration compared to normal cells at isotonic osmotic pressure. Moreover, we could promote or suppress CPP's penetration to erythrocytes under hypotonic or hypertonic conditions, respectively^9,10,13^ (Supplementary Fig. S4^10^). As a result, we succeeded in controlling CPP cytolysis by osmotic pressure as a physical stimulus^9,10,13^. To our best knowledge, this is the first successful attempt to control the cytolysis of CPP by membrane curvature modulation.

In order to confirm the second hypothesis further, we chose a giant unilamellar vesicle (GUV) as a model cell membrane. We have investigated the effect of osmotic pressure on the penetration of CPP to the GUV made of egg yolk phosphatidylcholine (EPC). GUV formation was confirmed by differential interference contrast microscope (DICM) observation. The size distribution was measured by dynamic light scattering (DLS), as shown in Supplementary Fig. S5.

**Figure 3:**
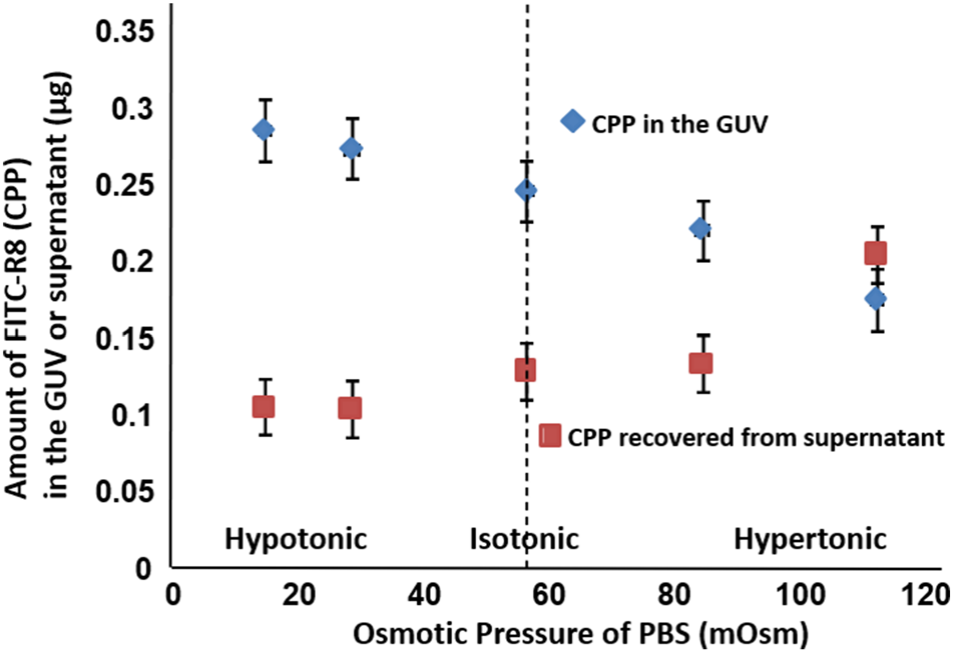
Effect of Osmotic pressure for the Cytolysis of FITC-R8 (CPP) into GUV made of the egg yolk phosphatidylcholine (EPC) (Lipid/peptide molr ratio (L/P) = 1000, 10 min, at 37 °C, n = 5).

Cytolysis of FITC-R8 to EPC GUV was conducted similarly to erythrocyte by changing the osmotic pressure, as shown in Fig. 3. The amount of cytolysis of FITC-R8 increased by reducing the osmotic pressure, corresponding to the curvature increase, which is in good agreement with the results obtained for erythrocyte^9–11,13^. The recovery of FITC-R8 from supernatant inversely decreased by osmotic pressure reduction (Fig. 3). Thus, this experiment's reliability was confirmed by the total recovery rate of applied FITC-R8, as shown in Supplementary Table T1.

**Figure 4:**
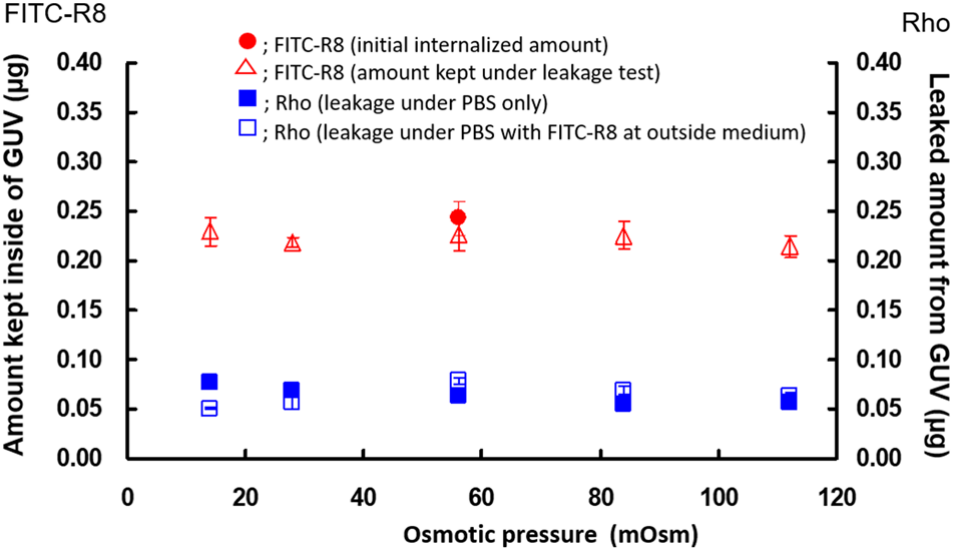
Effect of Osmotic pressure for the leakage of internalized FITC-R8 (CPP) and rhodamine (Rho) in the GUV (EPC, L/P = 1000, 10 min, 37 °C, n = 5).

Under the tested conditions, FITC alone could not permeate through the membrane into the cytosol regardless of osmotic pressure change^10^. The GUV membrane's barrier function remained intact as the leakage of rhodamine pre-encapsulated in the GUV stayed very low and consistent under different osmotic pressures neither in the presence nor absence of FITC-R8 in the outer medium (Fig. 4). Furthermore, pre-internalized FITC-R8 could not get out of the GUV regardless of the osmotic pressure as shown in (Fig. 4). These results reasonably support the two hypotheses since the inner lipid membrane has negative curvature, preventing phase transition to Mesh_1_.

**Figure 5:**
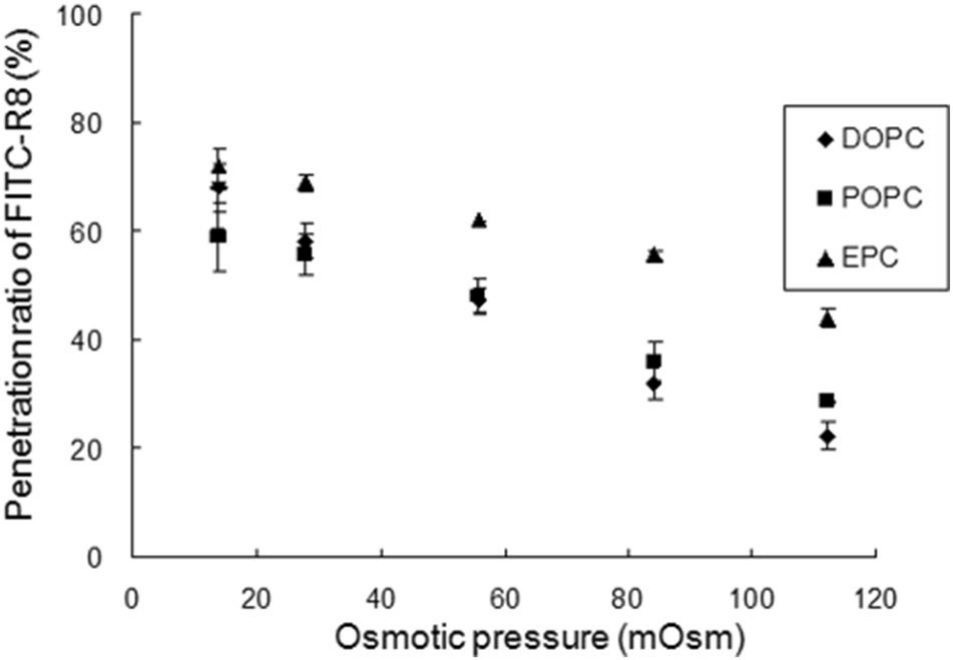
Effect of Osmotic pressure on the cytolysis of FITC-R8 (CPP) to the GUVs with varieties of Phospholipids (DOPC,POPC and EPC) (L/P = 1000, 10 min, 37 °C, n = 5).

**Figure 6:**
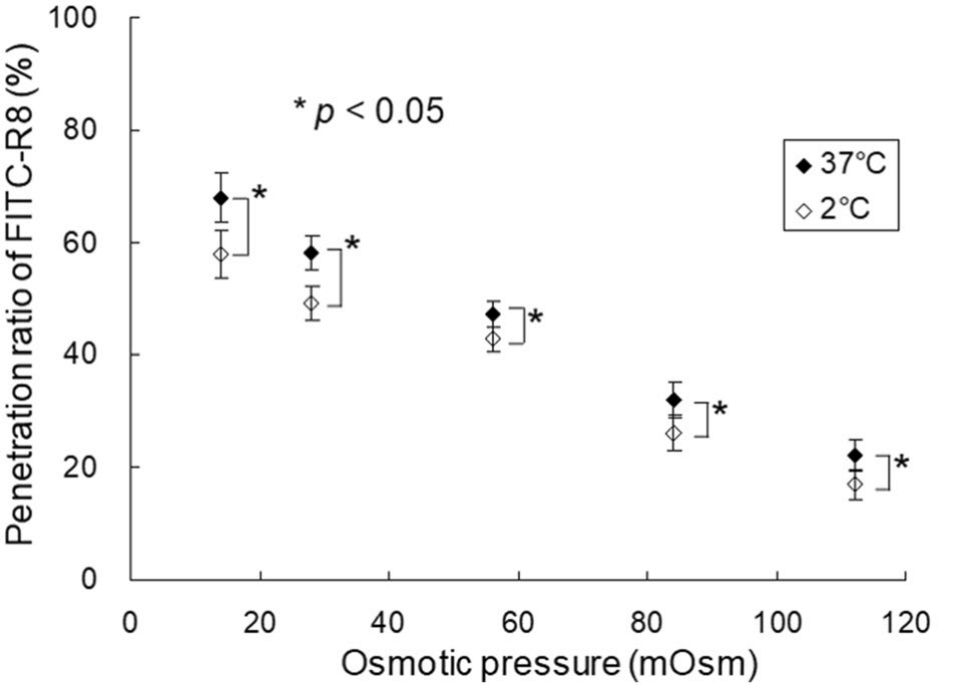
Effect of temperature on the cytolysis of FITC-R8 into EPC GUV under osmotic pressure change (L/P = 1000, 10 min, at 2 or 37 °C, n = 5, Significant differences were obtained using t-test).

Next, we have confirmed that osmotic pressure-dependent cytolysis of CPP is typical for GUVs composed of different varieties of phospholipids such as EPC, Dioleoyl-phosphatidylcholine (DOPC), and palmitoyl-oleoyl-phosphatidylcholine (POPC) as shown in Fig. 5. The cytolysis level is higher for EPC which is natural lecithin, as a mixture of various fatty acid residues. In terms of the effect of temperature, the penetration efficiency of FITC-R8 to DOPC was slightly low at 2 °C with statistical significance compared with that at 37 °C at all osmotic pressures (Fig. 6). This result indicates that membrane flexibility affects the FITC-R8 permeability, as we have previously reported. Namely, we found no cytolysis of FITC-R8 to SOPC GUV at 0 °C under confocal laser scanning microscope observation^11^. This is because SOPC has Tc at 6 °C and SOPC GUV is under gel state as hydrated solid at 0 °C.

These results support the cytolysis mechanism we have proposed and suggest controlling it by membrane curvature modulation. We have demonstrated it for fibroblast and keratinocyte by incorporating solutes instead of osmotic pressure to control CPP cytolysis^9,11^. Hoffmeister Effect of solute^12^ is one of the known phenomena to control the hydration level of self-assembled amphiphiles. Chaotropic solutes (water structure breakers) such as sodium thiocyanate (NaSCN) and 1,3-butanediol (1,3-BG) make the membrane curvature positive. On the other hand, kosmotropic solutes (water structure makers) such as sucrose make the membrane curvature negative^14,15^. Chaotropic solutes, such as NaSCN and 1,3-BG enhanced cellular uptakes of FITC-R8 without interfering with cell viability for dermal fibroblasts. On the other hand, sucrose, kosmotropic solute, suppressed CPP uptake6,9. Here, we have confirmed these solute effects for FITC-R8 cytolysis for GUV as well.

The uptake of FITC-R8 to the GUV was measured by the same procedure as the osmotic pressure change, but inside is pure water instead of PBS and the outer media was changed to an aqueous solution of NaSCN, NaCl and Na_2_SO_4_ at a concentration of 1700, 170 and 17 mM respectively. The translocation of FITC-R8 was c.a. 100 (µmol/mol lipid) for NaSCN, c.a. 50 (µmol/mol lipid) for NaCl and c.a. 20 (µmol/mol lipid) for Na_2_SO_4_. The order of FITC-R8 translocated to GUV was NaSCN > NaCl > Na_2_SO_4_, which follows the order expected by the Hoffmeister effect. As another example of curvature control for the cytosolic CPP translocation, Futaki et al. reported the incorporation of a hydrophobic moiety which interacts with the lipid membrane to induce positive curvature and proposed a loosening of lipid packing mechanism which would be a key factor of membrane translocation of CPP^15^. Examples are an ion-pair formation of Rx with pyrenbutyrate^16^, an introduction of hydrophobic moiety by acylation of Rx^17^, or incorporation of curvature inducing peptide^18^.

**Figure 7:**
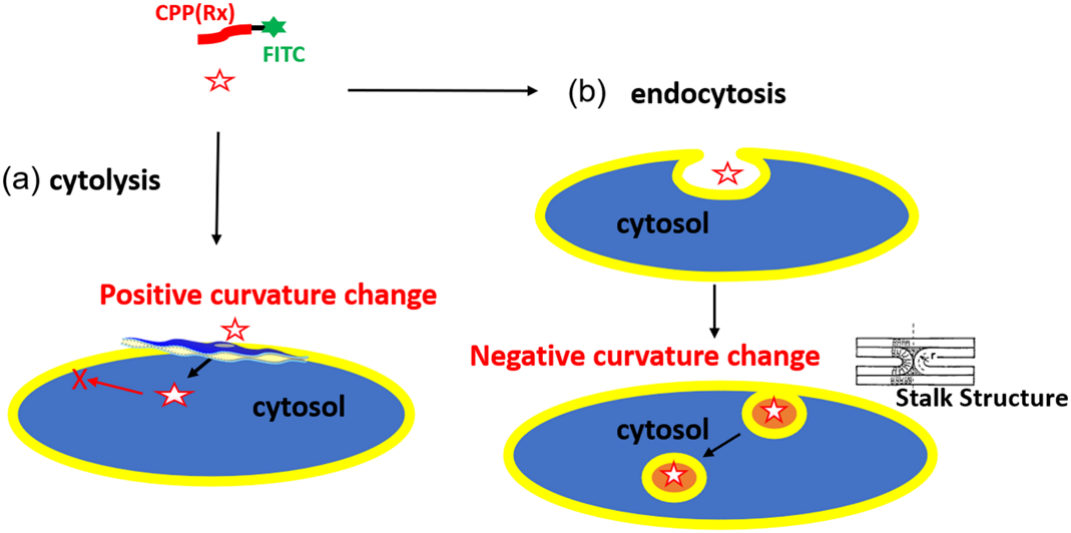
Schematic model of CPP permeation into cells. (**a**) Direct cytosolic (Cytolysis) internalization by positive curvature generation as local and temporal phase change to Mesh_1_, (**b**) Endocytic internalization (Endocytosis) by negative curvature generation through Stalk structure. Under the Cytolysis mechanism shown here, internalized CPP would not leak out because inner lipid membrane has negative curvature and oppose for the phase change to Mesh_1_.

Contradictory to the positive curvature generation, Mirshra et al. proposed the formation of holes as the catenoid structure with saddle-splay negative Gaussian curvature to explain the internalized leakage of the fluorescent probe inside of GUV caused by R6-FITC^19^. Then they showed phase transfer of Lα composed of dioleoyl-phosphatidylethanolamine (DOPE) and dioleoyl-phosphatidylserine (DOPS) to V_2_ and further to H_2_^20^. In this case, the mean curvature of lamellar membrane changes to negative because both DOPE and DOPS have a smaller hydrophilic group (smaller ***a***) than DOPC***,*** resulting in ***SP*** increase. During this phase change, the transition of Mesh_2_ can be formed which has hyperbolic surfaces with holes as catenoid saddle-splay negative Gaussian curvature as well. Such reverse curvature generation to V_2_ and H_2_ only happens through the Stalk structure^21^. The Stalk structure is a well-accepted model for cell fusion or fission processes and endocytosis (Fig. 7b).

Consequently, local and temporal positive curvature change is a crucial mechanism to make CPP penetrate directly into the cytosol (Fig. 6). Nature has systems to prevent fatal pore formation by clogging the pores with large membrane protein or creating local lipid domain (raft) (as shown in Supplementary Fig. S7)^22^. Local adsorption of CPP would be too quick for such a defense system to respond.

While we would not exclude the importance of the Stalk model to explain CPP’s penetration, but this path would be generated by negative curvature to lead to the formation of endosome as discussed (Fig. 7). There were no endocytosis uptakes observed in the experiments reported here.

## Conclusion

In conclusion, local and temporal positive curvature change seems crucial to promote the CPP cytolysis. With this knowledge, controlling the translocation of CPP by physical or chemical stimuli should become a promising way for both the development of advanced DDS and the prevention of CPP’s invasion.

The mechanism proposed here could be expanded to other phenomena and applications as a general mechanism to control the bi-layer membrane’s structural change. One possible example may be the permeation of cationic nanoparticles through the cell membrane since adsorption of cationic nanoparticles to the phosphate anion of planar and flexible lipid membrane would increase the cross-sectional area ***a***. which increases the curvature (decrease **SP**) to generate local and temporal Mesh_1_ structure. Utilization of CPP or any material and surrounding condition to induce positive curvature could prevent the formation of the Stalk intermediate. Although these assumptions must be experimentally proven, utilization of this proposed mechanism as a temporal and local curvature modulation would open up a novel way to combat membranous malfunctions.

## Materials and methods

### Materials

Dioleoyl-phosphatidylcholine (DOPC), palmitoyl-oleoyl-phosphatidylcholine (POPC), and egg-yolk phosphatidylcholine (EPC lipids provided by NOF Co., Tokyo, Japan) were used for the preparation of GUV. Phosphate buffered saline (PBS, Wako Pure Chemical Co. Ltd., Osaka, Japan) was used as a solvent. Oligo-arginine (Rx, x = 4, 6, 8), used for the phase behavior of DOPC under equilibrium, were synthesized in the Fmoc-solid-phase manner at Imura laboratory. FITC-R8 (fluorescein isothiocyanate conjugated with *γ*-aminobutyric acid as a linker to N-terminus of R8, synthesized at Futaki laboratory) was used for penetration study as CPP. Rhodamine B (Rho, 98% pure; Kanto Chemical Co. Inc., Tokyo, Japan) was used as the leakage marker. Triton-X100 (TX100, Sigma-Aldrich Inc., Missouri, USA) was used for the destruction of liposomes to examine the penetration ratio of FITC-R8 and leakage ratio of Rho.

### Preparation of erythrocyte

The preparation method was conducted in the same manner previously reported^9^. 5 ml of preserved sheep blood (Kojin Bio Co. LTD., Saitama, Japan) was centrifuged (4 °C, 3000 rpm, 3 min) and washed with 5 ml of physiological saline and centrifuged (4 °C, 3000 rpm, 3 min). The same operation was repeated to obtain erythrocyte pellet as a precipitate. 400 μL of erythrocyte pellets was washed with 400 μL of 20% PBS (isotonic) and centrifuged. An erythrocyte pellet for the cytolysis assay was obtained after the repeated operation.

### Preparation of GUV

GUVs were prepared by the freeze-melting and natural swelling method^23^. Lipids dissolved in chloroform were placed into the vials, and then the solvent was evaporated. After being dried in vacuo for 16 h in the dark, lipid films were hydrated with 0.01 mg/mL of Rho/3 M KCl solution to give 20 mM lipid concentration. Lipid suspensions were sonicated for 30 min (CS-20, SHIBATA SCIENTIFIC TECHNOLOGY Ltd., Saitama, Japan), frozen using liquid nitrogen rapidly, and melted at room temperature. The suspension was vortexed for 1 min. This step (from freezing to vortexing) was repeated six times. It was transferred to a dialysis membrane (UC-8-32-25; Viskase Co. Ltd., Chicago, USA) and dialyzed against 20% PBS for at least two days at room temperature and, the dialysis solution was daily replaced by a new one. In this study, 20% PBS is defined as ‘isotonic solution’ and PBS solution at concentrations less than 20% as ‘hypotonic solution’ and at higher than 20% as ‘hypertonic solution’.

### Measurement of particle size distribution and microscopic observation of GUV

GUV’s particle size distribution was measured with a dynamic light scattering measuring apparatus (DLS, Nicomp 380ZLS, Agilent Technologies, Tokyo, Japan) using an argon laser (532 nm). For the observations of GUV prepared differential interference contrast microscope (DICM, ECLIPSE E-600 Nikon, Japan) was used.

### Small-angle X-ray scattering (SAXS) measurements

SAXS experiment was carried out using a SAXSess camera (Anton-Paar Co., Ltd., Graz, Austria) and a PW3830 X-ray generator (PANalytical Ltd., ALMELO, Netherlands) was operated at 40 kV and 50 mA. All samples were filled into a thin quartz capillary and set in a sample holder unit, which can control the temperature within 0.1 °C accuracy. (TCS120, Anton-Paar Co., Ltd., Graz, Austria). An imaging plate was used for recording the scattering data and read out by a Cyclone storage phosphor system (Perkin-Elmer Co., Ltd., Massachusetts, USA) to get scattering data.

### Penetration assay of CPP into erythrocyte

The penetration assay of CPP into erythrocyte was conducted in the same manner previously reported^9,10^. To the 400 μL of erythrocyte pellet, 400 μL of PBS at concentrations of 98 mM (196 mOsm as hypotonic), 140 mM (280 mOsm as isotonic), and 182 mM (364 mOsm as hypertonic) were added for the evaluation of cytolysis by CPP. 20 μL of 40 μM FITC-R8 was added in the 1.40 * 108 cells of sheep erythrocyte. After incubation for 10 min at 37 °C, the solution was centrifuged (4 °C, 12,000 rpm, 10 min) and washed with 400 μL of isotonic PBS and centrifuged (4 °C, 12,000 rpm, 10 min). After the repeated washing process, the precipitate was mixed with 200 μL of 10% TX-100 isotonic PBS to solubilize the membrane. FITC-R8 penetrated to erythrocyte was determined by fluorescence spectrometry (Ex = 495 nm, Em = 520 nm). All measurements were used five samples (n = 5), and a significant difference was calculated by using the t-test (*α value was fixed at 0.001 and *p* < 0.001, **α value was fixed at 0.01 and *p* < 0.01). Throughout these experiment, no hemolysis was observed within the range of osmotic pressure tested, which suggests that membrane barrier for hemoglobin in the erythrocyte was kept intact^9^.

### Penetration assay of CPP into GUV

Penetration assay of CPP into GUV was conducted in the same manner previously reported^23^. 600 μL of 20 mM GUV was centrifuged (4 °C, 10,500 rpm, 20 min) and washed three times with 20% PBS and 1200 μL of PBS at concentrations at 5, 10, 20, 30, and 40% (14, 28, 56, 84, and 112 mOsm) were added to precipitate and control the shape of GUV. The following experiments were done on separating five liposomal suspensions. That is, 200 μL of 10 mM GUV was mixed with 780 μL of each concentration of PBS and 20 μL of 100 μM FITC-R8. In this case, the molar ratio of lipid/peptide (L/P) was fixed at 1000. A total of 1 mL samples were incubated at 37 °C or 2 °C for 10 min in the water bath and centrifuged (4 °C, 10,500 rpm, 20 min) two times. The precipitate and 100 μL of supernatant were mixed with 200 μL of 10% TX-100 and 100% PBS to make the total volume 1 mL. The amount of penetrated FITC-R8 was analyzed using the fluorescence spectrometer (Ex = 490 nm, Em = 520 nm, RF5300PC; SHIMADZU Co., Kyoto, Japan). Similarly, Rho’s amount entrapped was analyzed using the same instrument as for Rho (Ex = 555 nm, Em = 575 nm). The ratio of penetrated FITC-R8 and Rho leakage ratio was calculated using the following equations:

1 $$P = \frac{{\text{A}}}{{\text{U}}} \times 100$$

2 $$L = \frac{{I_{{L{ - }Rho}} }}{{I_{{L{ - }Rho}} + I_{{NL{ - }Rho}} }} \times 100$$

In Eq. (1), *P* is the penetrated ratio of FITC-R8 ratio, *A* is the amount of penetrated FITC-R8 into GUV calculated using the standard curve, and *U* is the amount of FITC-R8 used in this experiment. In Eq. (2), *L* is the leaked Rho ratio, and *I*_*L-Rho*_ and I_*NL-Rho*_ are the fluorescence intensities of leaked and non-leaked Rho.

### Confocal laser scanning microscopic observations of GUV

A Leica TCS SP2 confocal laser scanning microscope (CLSM, Leica Co., Tokyo, Japan) was used for observations of GUV after penetration assay. The fluorescence detection was performed in the range 490–520 nm (FITC-R8) and 550–570 nm (Rho). The temperature was fixed at 25 °C.

## Supplementary Information

Supplementary Information.
