# Supplementary material for: Direct entry of cell-penetrating peptide can be controlled by maneuvering the membrane curvature
Source: Sci Rep. 2021 Jan 8;11:31. doi: 10.1038/s41598-020-79518-1 (PMC7794472; doi:10.1038/s41598-020-79518-1)
Supplement: Supplementary file 1 — Supplementary Information. [file 41598_2020_79518_MOESM1_ESM.pdf]

Supplementary Information (Figs S1-S7, Table T1)

Direct entry of Cell-Penetrating Peptide can be controlled by maneuvering the membrane curvature

Kazutami Sakamoto,*[1] Taku Morishita [1], Kenichi Aburai [1], Daisuke Ito [1], Tomohiro Imura [2], Kenichi Sakai [1], Masahiko Abe [1], Ikuhiko Nakase [3], Shiroh Futaki [3], and Hideki Sakai [1]

1Tokyo University of Science, 2641 Yamazaki, Noda, Chiba 278-8510
2The National Institute of Advanced Industrial Science and Technology
3 Institute for Chemical Research, Kyoto University, Gokasho, Uji, Kyoto 611-0011
*Corresponding author: kazusaka@rs.tus.ac.jp

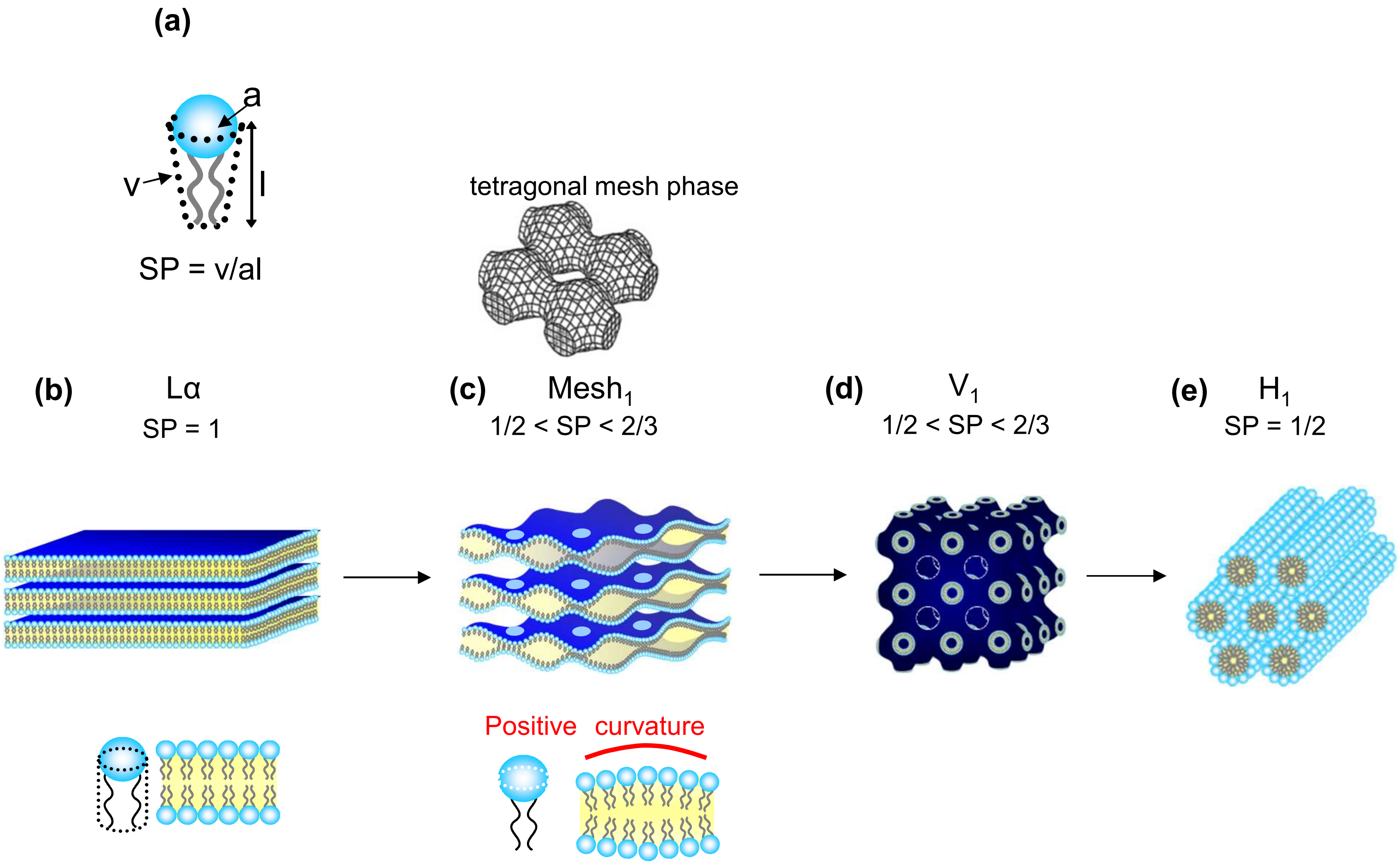

Supplementary Figure S1 General phase behavior of self assembled amphiphile[10-12]

(a) Surfactant parameter (SP = v/al : Equation 1), (b) Lα : lamellar phase, (c) $Mesh_1$: tetragonal mesh phase, (d) V_1 : bicontineous cubic phase, (e) H_1 : hexagonal phase.

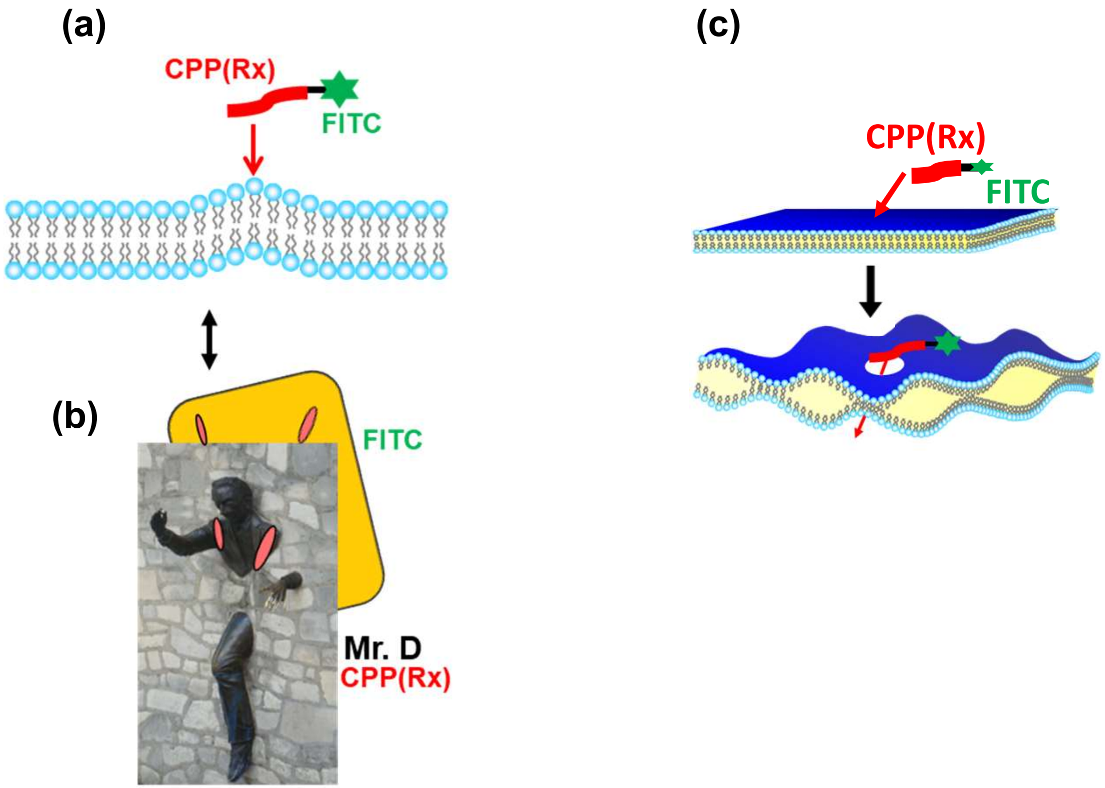

Statue at Montmartre, Paris
Courtesy of Dr. Nomura, Hokkaido Univ.

Supplementary Figure S2 Conceptual analogy of CPP permeation : The wall (Bio-membrane) changes to permeable fluid when Mr. D (CPP) comes to contact, then returns to solid barrier when he passed through

(a) electrostatic adsorption of Oligo-arginine (Rx) as CPP molecule, (b) Mr. D walks through the wall[13], (c) Direct internalization (Cytolysis) of CPP[6-8].

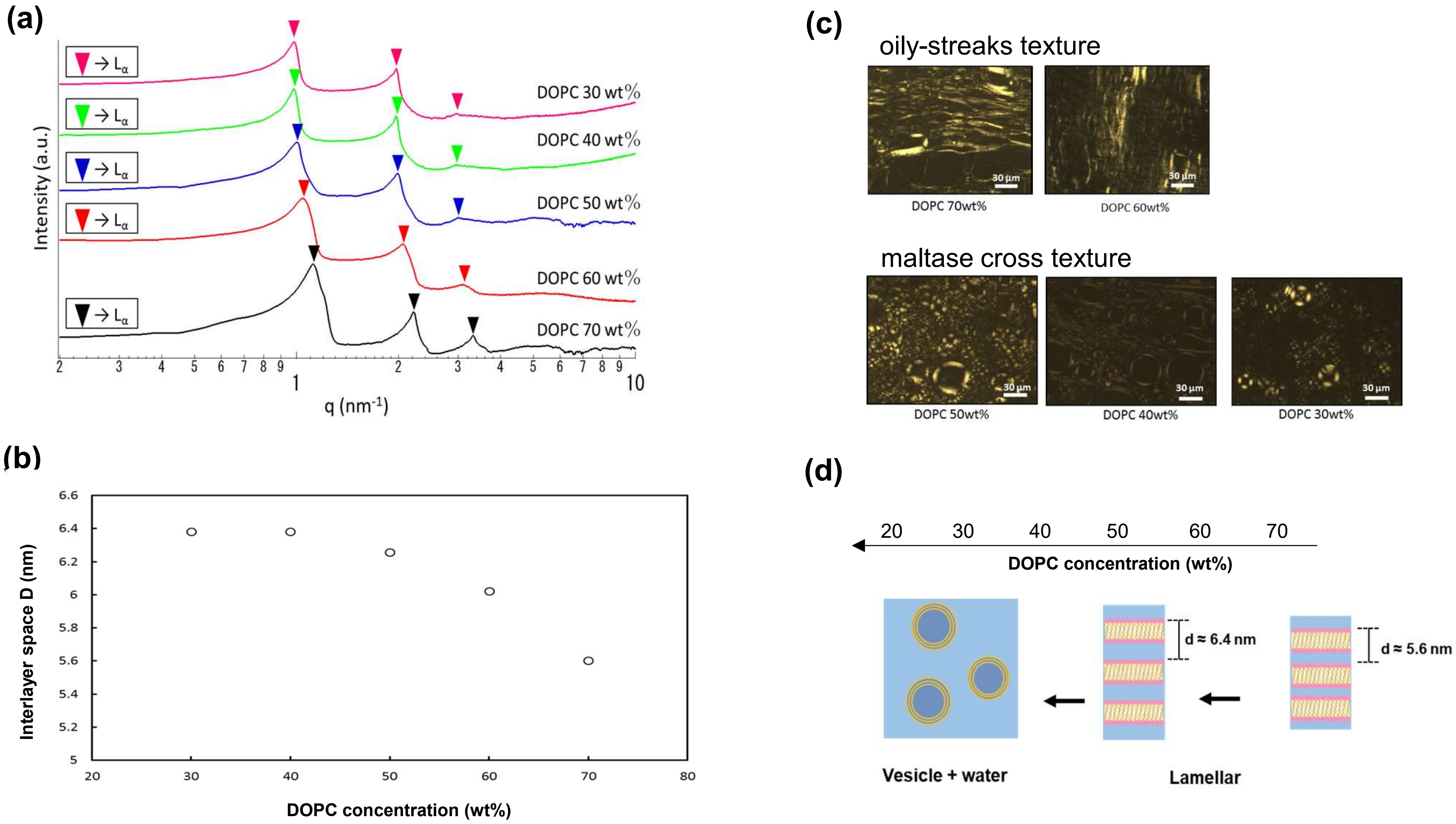

Supplementary Figure S3   Structure of lyotropic liquid crystal composed of DOPC and water
(a) Small angle Xray scattering (SAXS) spectra for DOPC/Water system at 25 ºC, q value of SXAS peaks for all the composition followed 1:2:3 ratio as Lα lamellar structure, **(b)** Interlayer spacing D (nm) calculated from the first peak q value, **(c)** Polarized microscope image of DOPC/Water Lα liquid crystal at 25 ºC , **(d)** Schematic diagram of DOPC /Water Lα liquid crystal based on the results shown in (a), (b) and (c)

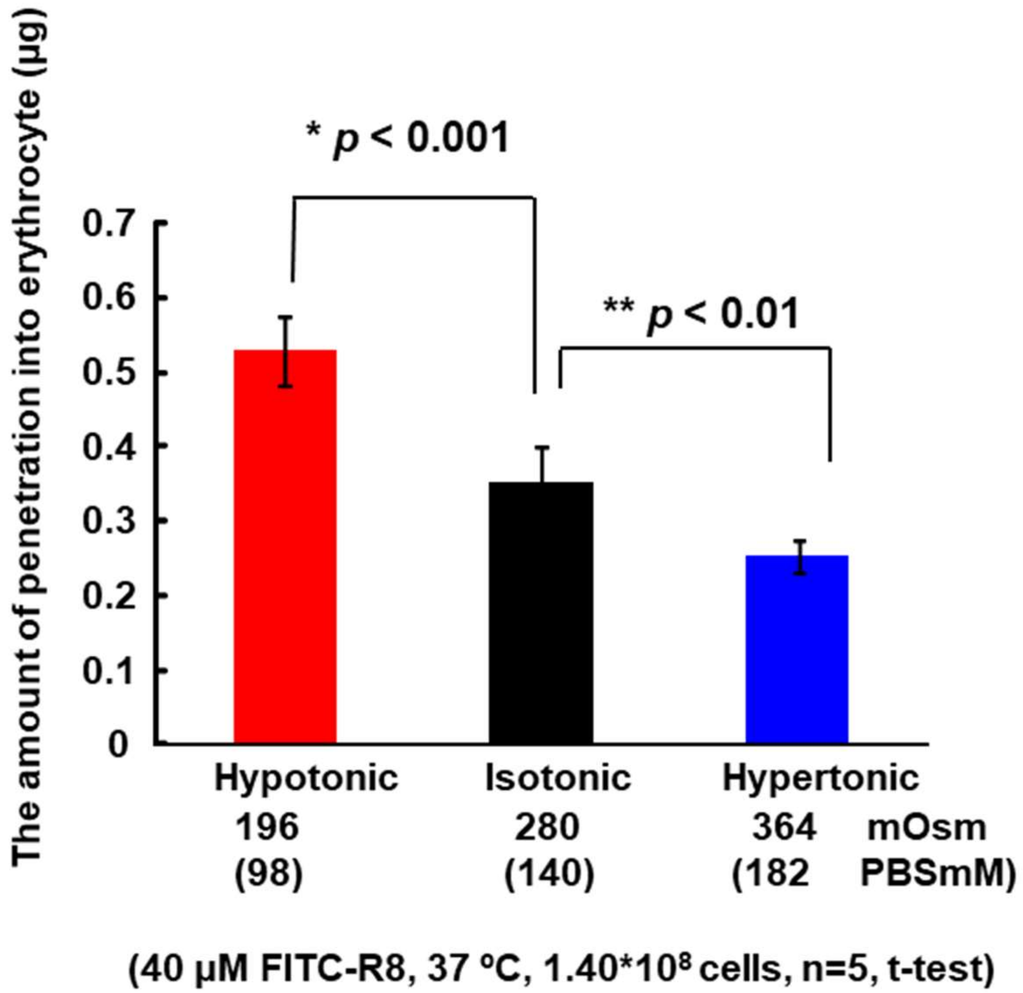

Supplementary Figure S4 Effect of the osmotic pressure on the penetration of CPP (FITC-R8) to the erythrocyte[8]

40μM FITC-R8 (20μL) , erythrocyte(1.40 * 10^8 cells) 10 min at 37 ºC, n=5

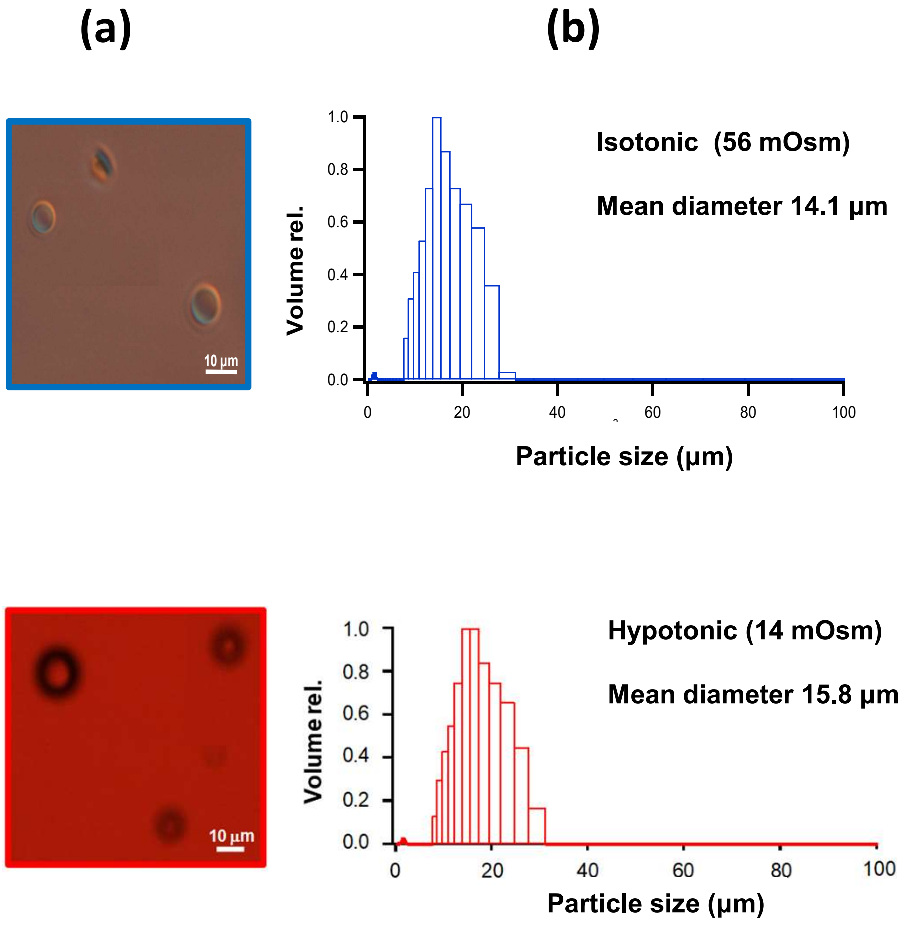

Supplementary Figure S5 Confirmation of GUV formation made of egg yolk phosphatidylcholine (EPC), and size distribution analysis
(a) Differential interference contract microscope (DICM) images,
(b) Particle size distribution analysis by dynamic light scattering (DLS)

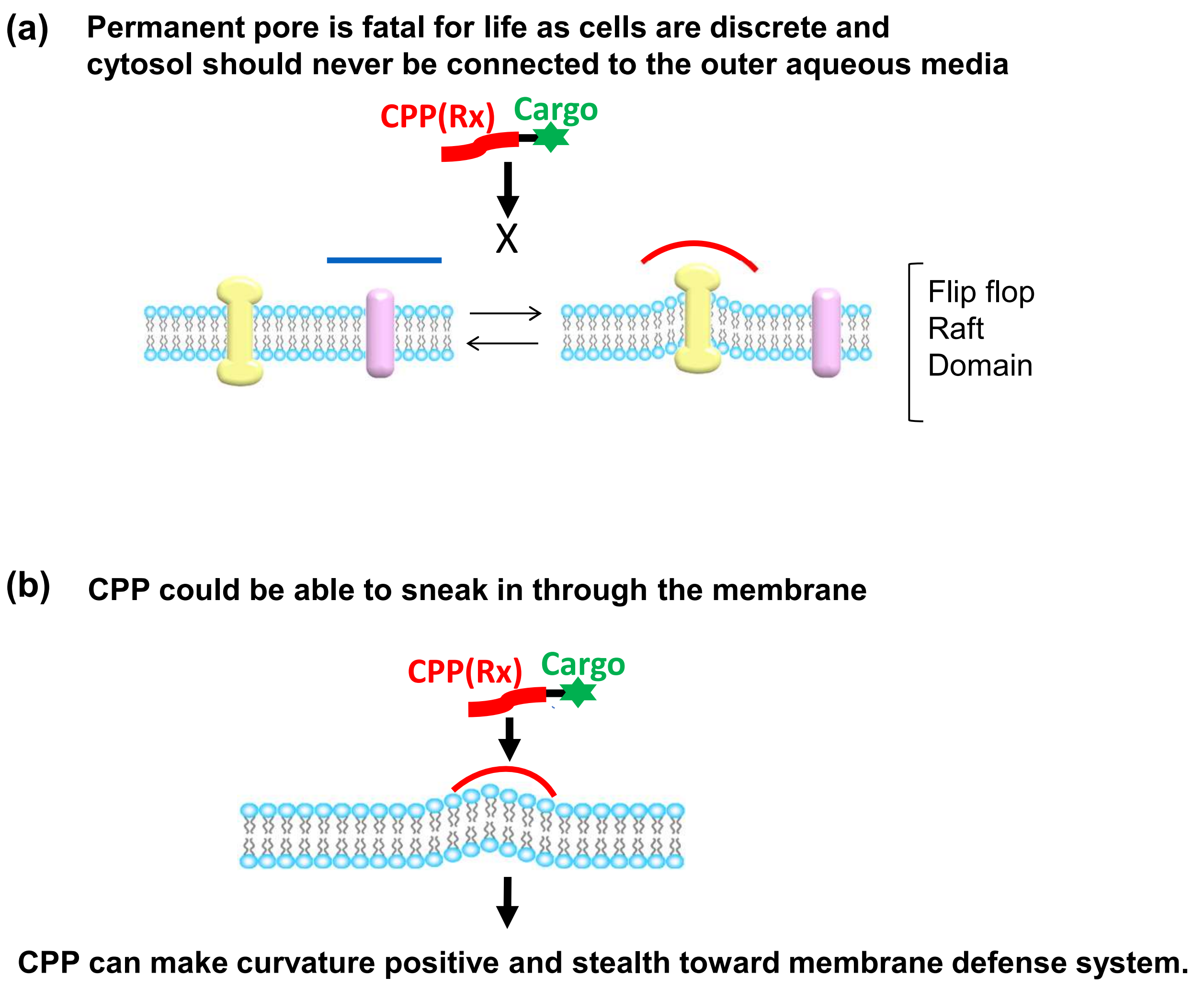

Supplementary Figure S6 Defense system in nature to prevent invasion of external hazardous matter through bio-membrane but CPP can sneak into cytosol

(a) Nature has systems to prevent fatal pore formation by clogging the pores with large membrane protein or by creating local lipid domain (raft) that are resistant to the pore formation[23], (b) CPP can permeate through membrane by cytolysis as local adsorption of CPP which would be too quick for the defense system to respond.

Supplementary Table T1. Material balance of FITC-R8 at the experiment on the CPP penetration experiment to EPC GUV

Osmotic pressure (mOsm)	Hypotonic 14	28	Isotonic 56	Hypertonic 84	112
Penetration (%)	74.2	71.0	63.8	57.3	45.4
Total recovery of FITC-R8 (%)	110.3	97.8	97.1	91.8	98.6

Material balance of FITC-R8 after the penetration study,
Lipid / peptide molr ratio (L/P) = 1000, 10 min, at 37 ºC, n=5)
